# Prevention and Treatment of Surgical Site Infections in Orthopaedic Surgery: An Italian Delphi Consensus on Risk Stratification and Wound Irrigation Strategies

**DOI:** 10.3390/jcm15051718

**Published:** 2026-02-24

**Authors:** Pier Francesco Indelli, Massimiliano De Paolis, Arcangelo Russo, Massimo Fantoni, Augusto Palermo, Giovanni Pomponio, Alessandro Scalise, Domenico Tigani, Bruno Violante, Steven L. Percival, Biagio Zampogna, Pierluigi Viale

**Affiliations:** 1 Südtiroler Sanitätsbetrieb, 39042 Brixen, Italy; 2 The Breyer Center for Overseas Studies, Stanford University in Florence, 50125 Florence, Italy; 3 Unità di Ortopedia e Traumatologia Ospedale S. Orsola, IRCCS Azienda Ospedaliero-Universitaria di Bologna, 40138 Bologna, Italy; massimiliano.depaolis@aosp.bo.it; 4 UOC Ortopedia e Traumatologia Ospedale Umberto I, Università Kore di Enna, 94100 Enna, Italy; 5 Dipartimento di Scienze Mediche e Chirurgiche, Fondazione Policlinico Universitario A. Gemelli IRCCS, 00168 Rome, Italy; 6 Chirurgia Protesica Anca e Ginocchio di Humanitas Gavazzeni Bergamo, 24125 Bergamo, Italy; 7 Clinica Medica, Ospedali Riuniti di Ancona, 60126 Ancona, Italy; giovanni.pomponio@ospedaliriuniti.marche.it; 8 Clinica di Chirurgia Plastica e Ricostruttiva, Dipartimento di Medicina Sperimentale e Clinica, Facoltà di Medicina e Chirurgia, Università Politecnica delle Marche, 60126 Ancona, Italy; 9 Unità Operativa Complessa di Ortopedia e Traumatologia dell’Ospedale Maggiore di Bologna, 40133 Bologna, Italy; d.tigani@ausl.bo.it; 10 Dipartimento di Ortopedia Centro di Alta Specialità di Chirurgia Protesica del Ginocchio e dell’Anca e Traumatologia dell’Ospedale Fatebenefratelli Isola Tiberina Gemelli di Roma, 00186 Roma, Italy; 11 Biofilm Centre, 5D Health Protection Group Limited, Liverpool L7 8XZ, UK; steven.percival@5dhpg.com; 12 Fondazione Policlinico Universitario Campus Bio-Medico, 00128 Rome, Italy; b.zampogna@policlinicocampus.it; 13 Research Unit of Orthopaedic and Trauma Surgery, Department of Medicine and Surgery, Università Campus Bio-Medico Di Roma, 00128 Rome, Italy; 14 BIOMORF Department of Biomedical, Dental, Morphological and Functional Images, University of Messina, Department of Orthopaedic and Trauma Surgery, A.O.U. Policlinico “G.Martino”, 98124 Messina, Italy; 15 Unità Operativa di Malattie Infettive, Ospedale Sant’ Orsola, IRCCS Azienda Ospedaliero-Universitaria di Bologna, 40128 Bologna, Italy; pierluigi.viale@unibo.it

**Keywords:** periprosthetic joint infections, PJI, debridement, polyhexanide, poloxamer, irrigation, SSI, surgical site infection, antiseptic solution, DAIR

## Abstract

**Introduction**: Surgical site infections (SSIs) and prosthetic joint infections remain among the most serious complications in orthopedic surgery, and chemical debridement is recommended for all septic revisions. The combination of polyhexanide (PHMB) and poloxamer (PLX), with in vitro antimicrobial and antibiofilm activity, represents a promising antiseptic solution. An Italian Delphi consensus was conducted to define the indications and clinical applications of PHMB/PLX as an antiseptic solution. **Materials and Methods**: A steering committee convened a panel of orthopedic surgeons, infectious disease specialists, and wound care specialists with expertise in musculoskeletal infections. A modified three-phase Delphi process was conducted. Twelve clinical questions and four outcome measures were developed through literature review and iterative discussion. Two Delphi rounds were conducted using a 9-point Likert scale, and statements were rated according to the GRADE method. **Results**: Twelve statements were developed, and all achieved strong consensus after two Delphi rounds. The panel identified key patient-related risk factors (smoking, diabetes, obesity, immunosuppression) and procedure-related risks (open fractures, primary/revision arthroplasty, prolonged operative time). Antiseptic irrigation was considered superior to saline, and PHMB-PLX was considered potentially useful based on expert opinion as an addition to mechanical debridement given its antibiofilm activity and good cytocompatibility. Low-pressure irrigation and short exposure times are the preferred application methods, while avoiding use on cartilage or neural tissues. **Conclusions**: The Delphi panel reached strong consensus supporting the intraoperative use of PHMB-PLX due to its potential as an antiseptic adjunct, supported by expert consensus and translational evidence for preventing and treating SSIs in orthopedic surgery. The panel recommended conducting high-quality clinical research to verify these findings and improve standardized irrigation protocols.

## 1. Introduction

Surgical site infections (SSIs) and periprosthetic joint infections (PJIs) remain the most feared adverse events in orthopedic surgery, causing significant morbidity and mortality along with a substantial increase in healthcare costs. For example, in a recent French study [1], PJI affected 1% of procedures, with an overall incidence of 11.6 events per 1000 patient-years, and led to a significant extension of hospital stays. In the same study, the in-hospital mortality hazard ratio was 12.01 (95% CI 10.63 to 13.57) for patients with SSIs after orthopedic surgery compared to those without SSIs. This increase in mortality was most significant among various types of surgeries analyzed (cardiac, digestive, etc.). The number of fracture-related infections (FRIs) is also steadily increasing [2], which is driven by increases in surgical volumes and antibiotic resistance. Therefore, preventing and managing musculoskeletal infections remains a primary focus for both clinicians and researchers. 

Since bacterial biofilms are among the most critical factors in promoting the onset of clinically relevant infections [3], interventions aimed at preventing and removing them are attracting significant interest from the medical community. In addition to mechanical debridement, irrigating the surgical wound with saline or antiseptic solutions is now standard practice in preventing and treating PJIs [4].

In addition to commonly used antiseptics such as povidone-iodine, new solutions, including sodium chloride/hypochlorous acid/sodium hypochlorite and oxychloride, with improved biofilm activity, have recently been introduced [5,6,7]. In addition, a combination of polyhexanide (PHMB), an effective and well-established antiseptic for treating chronic wounds, and poloxamer (PLX) (Preventia™, a brand of Paul Hartmann AG, Germany), a surfactant intensely active against bacterial biofilm, has recently been proposed as a promising new agent for wound irrigation, including in orthopedic surgery [6,7,8,9]. Unfortunately, randomized clinical outcome trials supporting the use of PHMB/PLM-based antiseptic solutions are lacking. While developing clinical practice guidelines is the preferable method to improve clinical management, they require a sufficient amount of reliable evidence. However, when the available evidence is of low of very low quality many experts base their decisions on consensus-based methods [10].

Due to these limitations, the authors decided to conduct a Delphi expert consensus involving a multidisciplinary panel of professionals with the goal of: (1) identifying potentially beneficial indications based on the available preclinical evidence and expert experience for the PHMB/PLX combination in prevention and treatment of SSIs; (2) establishing research priorities for future randomized clinical trials. This consensus primarily addresses wound irrigation strategies for high-risk scenarios involving infected implants (e.g., periprosthetic joint infections managed with DAIR procedures, infected revision arthroplasty, and contaminated open fractures), rather than comprehensive treatment protocols for all types of established SSIs. In this consensus, surgical site infections (SSIs) included FRIs after fracture fixation and PJIs after arthroplasty. These implant-associated infection subtypes merited separate consideration because of their unique biofilm-related pathophysiology and management challenges.

## 2. Materials and Methods

This project was initiated and developed by a multidisciplinary Organizing Committee (OC) composed of three specialists in infectious diseases, microbiology, and orthopedic surgery.

In January 2025, the OC assembled an interdisciplinary and geographically heterogenous panel consisting of five Italian orthopedic surgeons (from SudTirol, Lombardy, Emilia-Romagna, Lazio and Campania), one Italian plastic surgeon (from Marche), and one infectious disease specialist (from Lazio), all with demonstrated experience in research and clinical care for musculoskeletal infections (SSIs, FRIs, PJIs, spinal infections) in at-risk patients. One methodologist (GP) with specialized expertise in designing and conducting consensus initiatives was also enrolled for support.

For this project, a systematic approach primarily based on the modified Delphi technique was used, as recommended by the National Institutes of Health in 2009 (Consensus Development Program) and similar Italian National System for Guidelines, with adjustments to suit the topic of interest [11,12,13,14]. All 10 experts involved in the project (3 from the Steering Committee and 7 from the panel) participated as voters in the Delphi rounds.

### 2.1. Phase 1: Definition of the Problem, Questions, Literature Search, and Appraisal

The OC identified four key areas: 1. defining proper intraoperative management of the surgical wound (primary goal); 2. identifying the most significant risk factors for SSIs to pinpoint high-risk patients and procedures; 3. selecting pre- and post-surgery interventions to reduce the risk of infectious complications; and 4. determining which clinical and organizational outcomes are critical or at least necessary for assessing the impact of any preventive measures.

For each area, the OC specified particular clinical and organizational scenarios along with associated questions. This was accomplished through a structured face-to-face meeting, where the results of a preliminary literature search were compared with personal experience and values (EPICOT+ method) [14].

**Figure 1:**
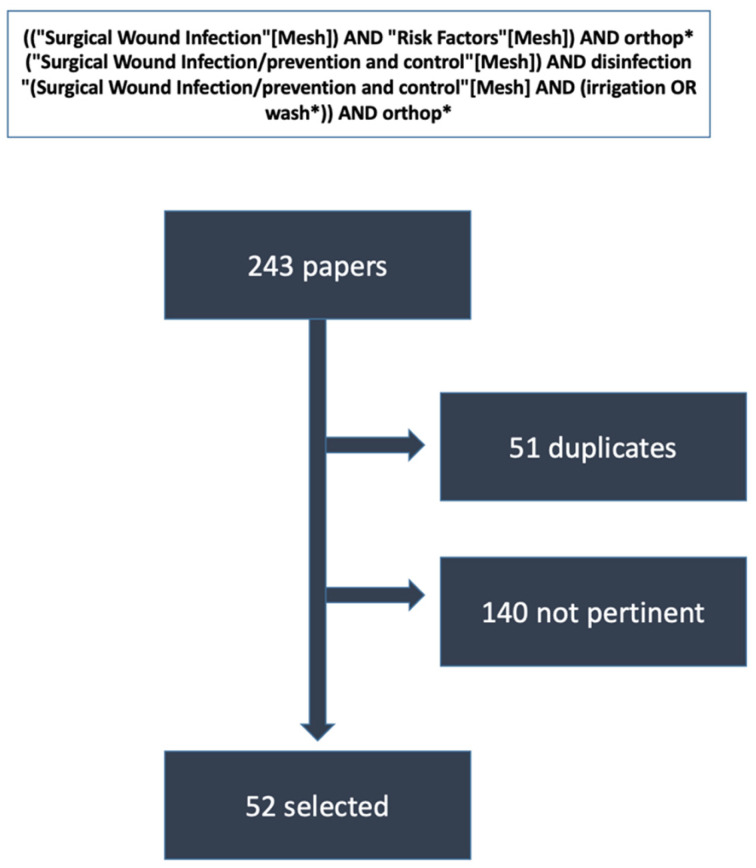
Results of the extensive, not systematic literature search. Limits: Clinical Trial, Consensus Development Conference, Guideline, Meta-Analysis, Observational Study, Randomized Control Trial, Systematic Review, Review, Adult: 19+ years, from 2015/1/1. * Mesh terms used during the literature search.

A final list of 12 questions and 4 outcomes was developed and approved. Afterwards, a comprehensive literature review was conducted to assess the current evidence on the topic. PubMed, Google Scholar, and Scopus were searched according to the strategies outlined in Figure 1, focusing on clinical trials and systematic reviews. 

The search was limited to the past 10 years. The same searches were repeated without using ‘Mesh’ to overcome limitations from an ongoing, incomplete indexing process where needed. Additionally, a manual search was performed through references of the selected articles. 

The last update was on 30 June 2025. The retrieved papers were evaluated for consistency and quality. The non-systematic nature of the review prevented assigning a formal level of evidence.

### 2.2. Phase 2: Statements, Consensus Development, and Measurement

Evidence reports and the approved questions were submitted to the expert panel in February 2025 during a plenary meeting. After structured and thorough discussion, 12 statements were proposed, along with notes and comments where appropriate.

The statements were collected to form a preliminary list, which was then presented to an initial voting round to evaluate the level of agreement among the panel and gather additional suggestions. After several updates and revisions, the statements underwent multiple Delphi rounds until agreement was reached on all statements or voting results stabilized for two consecutive rounds. A minimum of two rounds was planned in order to give the experts the opportunity to request minor modifications or additions to the text during the first voting round. Two rounds of anonymous voting were sufficient to reach a clear consensus on every statement, with minimal shifts in scores between the first and second rounds (see Table 1). Voting took place on a dedicated web platform that guaranteed complete privacy. An 80% agreement threshold was established for approving statements. Agreement was defined as a vote of ≥7 on the Likert scale. GRADE Working Group suggestions were used to assess the strength of agreement among the panelists by assigning the grade “strong” or “weak”. Specifically, a scale from 1 (no agreement) to 9 (strong agreement) was used. Interquartile ranges (IQR) and medians (M) were calculated to assess the level of agreement. A statement was defined as characterized by:

- ‘Strong agreement’ if the median was ≥8 and the lower end of the IQR was >5
- ‘Weak agreement’ if the median was 6 or 7 and the lower boundary of the IQR was ≥5
- ‘Disagreement’ if the median was less than 5 and the upper boundary of the IQR was ≤5
- ‘Uncertain’ in the remaining situations (median = 5; median > 5 but lower quartile < 5; median < 5 but upper quartile > 5)

The percentage of “Strong Agreement” was also calculated. A similar parallel process was followed for the four outcomes selected by the OC and discussed in the plenary meeting with the panel.

### 2.3. Phase 3: General Discussion and Statement Approval

The list of the 12 statements was discussed in a plenary meeting (July 2025) through structured discussion to verify and refine the wording and gather additional comments.

## 3. Results

The thorough literature search, followed by careful manual screening of the 243 articles retrieved, identified 52 publications relevant to the 12 questions drafted (Figure 1). Statements 1–4 defined patients and procedures at higher risk of infection; statements 5–7 discussed generalities on surgical wound irrigation; statements 8–10 reviewed and discussed rationale, indications, and technical advice on surgical wound irrigation with PHMB/PLX; and statements 11–12 focused on pre-and post-operative procedures and outcomes (i.e., patient preparation, surgical wound closure, and post-surgery management). Most statements received strong agreement from the expert panel (Table 1).

## 4. Discussion

According to the narrative review, the main outcome of this consensus was the recognition that the use of antiseptic agents for intraoperative wound irrigation serves as both a preventive and therapeutic measure against several types of SSIs. In fact, the panel of experts reached strong agreement on the technical aspects, especially the preference for antiseptic solutions over saline or antibiotics and for low-pressure techniques to reduce tissue damage and bacterial spread. This Italian Delphi consensus was a structured effort by experts to develop preliminary expert-opinion-based recommendations for intraoperative wound irrigation in orthopedic surgery and to clarify the role of PHMB–PLX in this context. The aim of the panel of experts was to present hypothesis-generating guidance that requires future clinical validation.

Despite ongoing advancements in perioperative infection control, SSIs and PJIs continue to be significant clinical challenges. A key outcome of this initiative was to establish recommendations for the use of PHMB–PLX and its proper application methods. The experts agreed that PHMB–PLX is especially effective in high-risk procedures—such as open fractures and primary and revision arthroplasty—where the presence of biofilm is most critical. Its application should include brief exposure times and gentle, low-pressure irrigation, avoiding prolonged pooling or contact with cartilage and nerve tissue. These recommendations align with the current understanding of PHMB–PLX’s dual mechanism of action, combining rapid antiseptic effects with surfactant-mediated biofilm disruption while maintaining favorable cytocompatibility.

Another significant contribution of this consensus is the methodological foundation it offers for future research. By identifying key clinical questions, critical outcomes, and standardized application methods, the panel established a foundation for designing strong, informative randomized controlled trials to evaluate the clinical effectiveness, tolerability, and effect size of PHMB–PLX irrigation. The main strength of this work is its rigorous and systematic approach: a clear understanding of the problem, transparent negotiation among evidence, experience, and expert values, and a measurable assessment of agreement. 

Statements 1 to 4 focused on risk stratification to define patients and procedures at higher risk of infection. Multiple systematic reviews and recommendations from international scientific societies [15] highlight the significant role of current smoking and diabetes in increasing the risk of SSI. The relative risk for active smokers following orthopedic surgery related to trauma or joint replacement is consistently estimated between 2.6 and 2.8 in recent systematic reviews [15,16,17], while the risk increase seems to be lower in the context of spinal surgery [18]. Diabetes is another significant factor that can increase the incidence of infections by more than two percentage points [19]: maintaining pre-operative blood glucose levels below 150 mg/dL reduces the risk of SSI by about half (OR 0.59, *p* < 0.001) [20,21].

Obesity is another well-studied risk factor [21,22], with an OR ranging from 1.9 to 2.7. However, the thresholds used to define it vary across studies, likely due to differences in reference populations (e.g., body mass index [BMI] > 24 kg/m^2^ in studies conducted in Asia versus BMI > 30–35 kg/m^2^ in Western countries). Nonetheless, very high BMI (>40 kg/m^2^) appears to be associated with an increased risk of deep-site or prosthetic joint infections [22,23]. A recent consensus report listed a BMI > 50 kg/m^2^ as a contraindication to arthroplasty [24]. Malnutrition is a less explored risk factor. However, evidence of its significance in increasing the risk of SSIs has been thoroughly evaluated in other consensus initiatives [23] and confirmed in recent reviews [25,26].

In oncological surgery, prior irradiation also appears to increase the risk of infection [27,28], although some reports are inconsistent [29]. Fewer, but more controversial, data support other patient-related risk factors, as endorsed by the experts’ opinion, due to the scarcity of dedicated studies and flaws in experimental design (mostly post hoc or secondary analyses). 

Regarding procedure-related risk factors, the systematic reviews cited and other studies emphasize the increased risk linked to open fracture surgery and implantation of prosthetic material. Surgery on already infected areas, including oncological procedures—especially when near colonized tissues like the pelvis—is also universally considered at high risk for infectious complications. The risk of SSIs when revising infected surgical wounds or implants is also higher, with a tendency for lower risk in one-step procedures compared to two-step or DAIR [30,31,32,33]. The risk increase seems considerably lower when repeated procedures are done on non-infected areas [34]. Another relevant procedure-related risk factor is the prolonged duration (>60 min) of the surgical intervention [35], which often correlates with longer procedures, usually in complex cases, and may involve extensive exposure and significant tissue damage [36].

Although some inconsistencies exist in the literature, mainly due to the retrospective design and heterogeneity across various aspects of the available studies, there was little doubt among the panel of experts about the importance of the patient-related risk factors listed in statement #1. Biological plausibility and clinical experience strongly support this. Current smoking, poorly controlled diabetes, and obesity are the most significant factors. Notably, a BMI over 50 kg/m^2^ should be regarded as a strong contraindication for elective joint replacement surgery. There was also strong agreement on the importance of immunosuppression [37], as well as on the potential impact of superficial skin fragility, such as that seen in patients on chronic steroid therapy, recurrent soft tissue infections, or changes in skin vascularization. Although it is not listed in the statement because establishing it as an independent risk factor is difficult, significantly advanced age should also be considered an additional negative prognostic factor. 

A debate emerged about how much importance should be assigned to different procedures. Specifically, the discussion centered on procedures used during the revision of infected areas. However, all experts agreed that choosing the proper surgical technique in cases of infected arthroplasty (DAIR vs. one-step vs. two-step) [38,39,40] was more crucial than assigning a specific risk level to each procedure. One of the key factors influencing risk of SSIs and PJIs is the extent of tissue damage caused by trauma or surgical procedures, especially when tissue mobilization disrupts normal blood flow to the area. For these reasons and because of their effect on operative duration, large surgical wounds should be considered at higher risk of infection.

Regarding open reduction internal fixation (ORIF) procedures performed to treat closed fractures, listed as statement #2 among low-risk procedures, the panel noted an exception for patients with polytrauma or fractures caused by high-energy trauma. The delay between fracture and surgery, especially in specific types of interventions such as femoral fractures, was identified by all the experts as an additional risk factor. Moreover, there was a strong consensus about the potential impact of the need for transfusions [41] and for patients passing through an intensive care unit, due to the increased risk of colonization with multidrug-resistant bacteria. Finally, the experts chose to adopt the CDC definition for superficial and deep surgical site infection [42], even though it is quite broad and not easily applicable to orthopedic surgery. The definition of PJI is more debated, and the panel chose to reference the findings of the international Consensus Meeting on PJI held in Istanbul in spring 2025.

Statements 5–7 discussed general principles of surgical wound irrigation. Several recent systematic reviews [43,44,45] confirmed that alcoholic chlorhexidine (CHX) was more effective than iodine-based preoperative skin antiseptics in reducing the risk of SSI across various surgical procedures, especially in orthopedic surgery. In the latest meta-analysis [45], the CHX group had a lower overall incidence of postoperative surgical site infections than the iodine group (RR = 0.30, 95% CI = 0.20–0.46, I2 = 95%, *p* < 0.00001). It showed similar effectiveness across different surgical procedures, as indicated by an RR of 0.25 [95% CI 0.15–0.41], I2 = 51%, and *p* < 0.0001 for general surgery; for cesarean sections, RR = 0.47 [95% CI 0.32–0.67], I2 = 82%, *p* = 0.0002; and for additional surgical procedures, including orthopedic surgery and others, RR of 0.47 [95% CI 0.34–0.65], I2 = 76%, with *p* < 0.00001. These findings are consistent with other similar systematic reviews and network meta-analyses [44,45]. In 2020, a randomized clinical trial (RCT) [46] was conducted to examine the preoperative use of CHX or PVP-I in lower limb trauma surgery: logistic regression analysis showed that the odds of wound healing complications were 3.5 times higher with PVP-I than with CHX (odds ratio = 3.5; 95% confidence interval, 1.1–11.2; *p* = 0.032). However, less robust retrospective studies have not always confirmed these results [47].

Multiple systematic reviews have examined the effectiveness of surgical wound irrigation in preventing SSIs and PJIs, comparing various aqueous antiseptic solutions with antibiotic solutions, saline, or no-irrigation strategies. Antiseptic solutions are more effective than saline or no irrigation in preventing SSI across general surgery and various surgical subtypes. A recent meta-analysis of 41 RCTs shows an OR of 0.72 (CI 0.57–0.93) for SSIs compared to saline [48]. The findings align with evidence from systematic reviews conducted on both general [49] and orthopedic [50] surgery. Some recent studies have challenged the superiority of antiseptic irrigation over saline [51,52]. However, these findings should be interpreted with caution as they are based on post hoc analyses of observational data, even when derived from registry sources. The majority of RCTs and meta-analyses show that antibiotic-based solutions have effects similar to those of antiseptics [51]. Nevertheless, concerns about their possible role in promoting colonization by antibiotic-resistant organisms have led most authors to advise against their routine clinical use [52].

Statements 8–10 reviewed and discussed the rationale, indications, and technical advice on surgical wound irrigation with PHMB-PLX in orthopedic surgery. PHMB-PLX irrigation provides a dual-mechanism approach: quick membrane-targeted killing (PHMB) and surfactant-driven EPS disruption (PLX). This combination decreases planktonic load and improves penetration into sessile biofilm bacterial niches found in orthopedic implant surgery. 

Robust in vitro and translational models demonstrate multi-log reductions in both planktonic and biofilm populations. Furthermore, evidence from ex vivo and murine translational models supports their application on implant surfaces such as titanium and hydroxyapatite coatings [53,54]. Few authors [55] have reported rapid planktonic killing of orthopedic-relevant bacteria by PHMB–PLX, with a 3–5 log_10_ reduction in CFU within 30–120 s at the experimental challenge concentrations used in bench studies (0.05–0.1% PHMB). Several studies [53,54,55] report minimum biofilm eradication concentration (MBEC) or MBEC-like endpoints for PHMB, which are slightly higher than planktonic MICs, as expected. In practical irrigation use, high local concentrations and repeated mechanical action (pulsatile lavage) help achieve effective local exposures without systemic toxicity. Cytotoxicity studies [56] also showed that short exposures (seconds to a few minutes) at concentrations that are effective against bacteria maintain cell viability of 60–80% in many assays, resulting in a favorable therapeutic index (ratio of antimicrobial effect to host–cell toxicity).

Nonetheless, it is recommended to avoid prolonged pooling on cartilage, exposed nerve tissue, or sensitive intracapsular structures. Additionally, preventing ad hoc increases in concentration or unvalidated dilution changes is recommended. While translational data are compelling, animal and ex vivo human tissue models are approximations: human surgical sites involve complex host immune and perfusion factors that may influence antiseptic kinetics and outcomes.

A few experts have effectively used PHMB-PLX in clinical practice [9]. Its key benefit is its promising ability to prevent and remove biofilm from foreign materials and tissues. In fact, evidence from wound care studies has shown that removing biofilm is crucial to achieve a favorable prognosis, whether in late or early (<4 weeks) surgical site or periprosthetic joint infections [57]. Additionally, using a ‘chemical debridement’ as an adjunct to mechanical debridement provides benefits in treating surgical wounds with limited space and hard-to-reach recesses [4]. Additionally, an important factor to consider is the proven effectiveness of PHMB-PLX at low pressure. In fact, PHMB-PLX (Preventia™) has been approved by regulatory agencies for use with low-pressure techniques. Of note, low pressure should be preferred in traumatic surgery, especially for open fractures [58]. 

Furthermore, the scientific and clinical community is increasingly moving toward establishing a contraindication for high-pressure intraoperative lavage in prosthetic surgery due to the risk of transferring pathogens from superficial tissues into the wound. The strength of the biological data on the effectiveness of combining a disinfectant and surfactant in reducing the need for extended contact times with surgical wounds and foreign materials led the authors to strong consensus on practical application methods, detailed in recommendation #10; nevertheless, the authors acknowledge that the recommended contact times (1 min in a sterile environment and 3 min in a contaminated environment) represent expert opinion extrapolated from preclinical data. The use of multiple antiseptics, including PHMB-PLX, has also been recently recommended by experts at the 2025 ICM on PJIs [4] for DAIR scenarios. Finally, in the panel’s unanimous opinion, the demonstrated low tissue toxicity, particularly concerning cells involved in repair processes, is another major strength of this disinfection strategy. 

However, all experts agree that the main barrier to the broader use of PHMB-PLX in orthopedic surgery is the lack of clinical studies. Developing high-quality randomized controlled trials to evaluate their effectiveness in preventing and treating surgical site infections in orthopedics should be a top priority for researchers.

Statements 11–12 focused on preoperative and postoperative procedures and final outcomes (i.e., patient preparation, surgical wound closure, and postsurgical management). Although providing recommendations on pre- and post-operative procedures was not the primary focus of this project, the expert panel chose to draft statements to help guide the future development of reliable and informative clinical trials on the use of PHMB-PLM in orthopedic surgery. The review by Siedelman et al. on surgical site infection prevention [59] and the 2021 clinical guidelines by the Italian Society for Orthopedics and Traumatology [60] served as the primary sources of evidence. After thorough discussion, a strong consensus was achieved on the 5 pre-operative procedure recommendations summarized in statement #11, as well as on the 4 post-operative recommendations listed in statement #12. Surprising results from a recent umbrella review [61] showed that intrawound vancomycin significantly reduces infection rates in primary joint arthroplasty, including periprosthetic joint and superficial infections, without increasing wound complications. This finding sparked a lively debate among the panel members because the ICM 2025 recommendations took a different direction [62]: ultimately, the prevailing consensus was to continue recommending against the use of vancomycin powder, in line with the findings of an international consensus.

During the discussion, a strong consensus emerged on the primary outcome to be tested in future trials focused on assessing the effectiveness of preventive or therapeutic interventions for infected surgical wounds in orthopedics. The presence of clinical signs and symptoms was unanimously considered the primary method to evaluate the outcome of interest, and a 90-day time point was selected in accordance with the 2024 National Healthcare Safety Network (NHSN) recommendations [63]; the 2-year minimum follow-up as a time to define the outcome of the intervention, specific to arthroplasty, was selected based on surveillance data from Sweden’s extensive national infection control program [64], which reported on the incidence of periprosthetic joint infection after primary total hip arthroplasty.

This consensus has several major limitations, making the recommendations invalid across all clinical scenarios for SSIs. The first limitation is the absence of a formal systematic review, which may have led to the omission of a few studies; however, the authors conducted a narrative review of the literature. Nonetheless, the project’s goal was not to create a formal guideline but only to produce preliminary expert-opinion-based recommendations, and it is unlikely that additional small or diverse publications would have significantly changed the experts’ consensus. Other major limitations include the small panel size, a single-country perspective (Italy), the use of a modified Delphi methodology with limited reporting on the between-round evolution of voting/statement refinement, the absence of cost-effectiveness analysis, limited reporting of patient-reported outcomes, and, especially, industry funding from a PHMB-PLX manufacturer. In general, however, it should be emphasized that the primary limitation remains the lack of clinical trials on PHMB–PLX, which should be addressed in future studies. To partially fill this gap, a U.S.-based survey on the intraoperative use of various wound irrigation systems has recently been published [54]. 

## 5. Conclusions

This Delphi consensus offers preliminary, expert-opinion-based recommendations for the intraoperative use of antiseptic irrigation systems in SSI scenarios, with a particular focus on PHMB–PLX and provides a template for future clinical research to measure its true clinical benefit. More studies are needed that focus on patients at high risk for postoperative SSI or on those undergoing implant-saving procedures in FRIs and PJI scenarios. Little is also known about the efficacy of various antiseptic solutions when surgeons debate whether to perform a single-stage or a two-stage revision for a periprosthetic joint infection. In the meantime, the multiple preliminary recommendations presented in this consensus are intended to support clinical decision-making in selected high-risk scenarios and in selected FRIs and PJIs while higher-quality evidence on how modern antiseptic irrigation solutions may affect SSI outcomes is generated.

## Figures and Tables

**Table 1:** Statements and results of voting (SA = Strong Agreement; WA = Weak Agreement).

#	Statement	Median	Q1–Q2	Grade
1	Current smoking and non-compensated diabetes are the major risk factors for SSI. Other relevant risk factors are: BMI > 35 kg/m^2^, malnutrition, immunosuppression, previous irradiation of the surgical site, previous infection involving the site of intervention, history of relapsing soft tissues infections and intra-articular therapy in the 3 months prior the surgery	8	8	SA
2	Procedures performed for the following should be considered at high risk for SSI: osteomyelitis infected prostheses (DAIR, one-step and two-steps procedures) grade 2 or 3 open fractures oncological surgery Procedures performed for the following should be considered at intermediate risk for SSI: closed fractures with implant of foreign material first prosthetic implant non-infected prostheses (i.e., prostheses revision) Procedures performed for the following should be considered at baseline risk level closed fractures without the implant of foreign material other elective interventions involving sterile territories	9	8	SA
3	Need to perform surgery under urgent or emergency conditions, large surgical wound site, prolonged duration of surgery further increase the risk of SSI	8	8	SA
4	In patients who underwent orthopedic surgery the definition of surgical site infection can be derived from the criteria provided by CDC	9	8	SA
5	In all patients, regardless of intrinsic risk level associated with patient-related and intervention-related factors, preoperative skin antisepsis using alcoholic CHX solution is recommended	8.5	8	SA
6	Surgical wound irrigation with antiseptic solution is preferable to irrigation with saline or no-irrigation in all interventions at increased risk of infection	8	7	SA
7	The use of antibiotic solutions for surgical wound irrigation should be discouraged due to the increased risk of inducing antibiotic resistance	9	8	SA
8	In all patients, especially in cases where at least one risk factor related to the patient or the type of surgery is present, the use of polyhexanide-poloxamer for surgical wound irrigation should be considered	8	7	SA
9	In high-risk orthopedic surgery, especially in cases where the surgery is conducted on infected territories, the use of polyhexanide-poloxamer for surgical wound irrigation is recommended	8	7.75	SA
10	When polyhexanide-poloxamer is used to irrigate surgical wounds: - it is recommended to use a volume of solution sufficient to completely fill the surgical site - to use a low pressure is preferable, particularly in traumatic surgery - the minimum suggested contact time is 1 min when the intervention is performed on sterile territories and 3 min when the intervention is performed on infected territories	8	7	SA
11	Panel recommendations for pre-operative procedures in all patients: Avoid razors for hair removal if not necessary. Clippers or depilatory cream can be used if hairs interfere with the site of incision Perform *Staph. aureus* nasal screening in all patients and decolonize if positive Recommend a pre-surgery bath with antiseptic soap or solutions Check blood glucose levels in the hours before the intervention and maintain glycemia < 150 mg/dL and HbA_1C_ < 8% Provide antimicrobial prophylaxis with weight-based antimicrobial agents selected based on most common pathogens for a specific procedure. The infusion of antibiotics must be completed not before 30–60 min from the intervention. Administer an additional dose if the surgery procedure lasts more than 3 h.	8	8	SA
12	Panel recommendations for post-operative procedures in all patients: Early SSI and/or PJI should be assessed at 90 days (first clinical evaluation after 48 h) Avoid antibiotic powder application to the patient’s skin or in the site of incision Use negative pressure wound therapy after open fracture treatment when immediate surgical wound closure is not appropriate Use negative pressure wound therapy as prevention in difficult to heal wounds and/or in presence of comorbidities such as diabetes, BMI > 30 kg/m^2^	8	7.75	SA
A	Appearance of clinical signs of infection at the intervention site within 90 days (early infection) or 2 years (prosthetic joints late infections) from the surgery	8	8	Critical
B	Appearance of systemic signs of infection * within 90 days from the intervention * (fever, increased C-reactive protein)	8	7.25	SA
C	Prolongation of hospital stay and/or need of systemic antibiotic therapy	8	8.75	SA
D	Need for re-intervention within 90 days	8	7.75	SA

## Data Availability

Data extracted during the Delphi Consensus process are available upon request to the corresponding author.

## References

[1] Foux L., Szwarcensztein K., Panes A., Schmidt A., Herquelot E., Galvain T., Phan Thanh T.N. (2025). Clinical and economic burden of surgical site infections following selected surgeries in France. PLoS ONE.

[2] Lourtet-Hascoët J., Bonnet E., Spera A.M., Ascione T., Chan M., Esposito S., Pagliano P., Scobie A., Ünal S., Giordano G. (2025). Fracture-Related Infections: Current Status and Perspectives from the International Society of Antimicrobial Chemotherapy. Antibiotics.

[3] Percival S.L., Emanuel C., Cutting K.F., Williams D.W. (2012). Microbiology of the skin and the role of biofilms in infection. Int. Wound J..

[4] Cashman J., Mortazavi S.M.J., Indelli P.F., Rele S., Haasper C., Yildiz F., Holland C.T., Lizcano J.D., Auñón-Rubio Á., Tai D.B.G. (2025). 2025 ICM: Debridement, Antibiotics, and Implant Retention (DAIR). J. Arthroplast..

[5] Honegger A.L., Schweizer T.A., Achermann Y., Bosshard P.P. (2025). Antimicrobial Efficacy of Five Wound Irrigation Solutions in the Biofilm Microenvironment In Vitro and Ex Vivo. Antibiotics.

[6] Zhou M., Liu Y., Fang X., Jiang Z., Zhang W., Wang X. (2024). The Effectiveness of Polyhexanide in Treating Wound Infections Due to Methicillin-Resistant Staphylococcus Aureus: A Prospective Analysis. Infect. Drug Resist..

[7] Watson F., Chen R., Saint Bezard J., Percival S.L. (2024). Comparison of antimicrobial efficacy and therapeutic index properties for common wound cleansing solutions, focusing on solutions containing PHMB. GMS Hyg. Infect. Control.

[8] Castiello G., Caravella G., Ghizzardi G., Conte G., Magon A., Fiorini T., Ferraris L., De Vecchi S., Calorenne V., Andronache A.A. (2025). Impact of Polyhexanide Care Bundle on Surgical Site Infections in Paediatric and Neonatal Cardiac Surgery: A Propensity Score-Matched Retrospective Cohort Study. Int. Wound J..

[9] Valpiana P., Salvi A.G., Festini Capello M.P., Qordja F., Schaller S., Kim J., Indelli P.F. (2025). Application of Molecular Diagnostics in Periprosthetic Joint Infection Microorganism Identification Following Screening Colonoscopy: A Case Report and Review of the Literature. Prosthesis.

[10] Djulbegovic B., Guyatt G. (2019). Evidence vs Consensus in Clinical Practice Guidelines. JAMA.

[11] Italian National Institutes of Health. https://www.iss.it/-/snlg-manuale-rbpca.

[12] Goodman C., Institute of Medicine (US) Council on Health Care Technology (1988). Medical Technology Assessment Directory: A Pilot Reference to Organizations, Assessments, and Information Resources.

[13] McMillan S.S., King M., Tully M.P. (2016). How to use the nominal group and Delphi techniques. Int. J. Clin. Pharm..

[14] Brown P., Brunnhuber K., Chalkidou K., Chalmers I., Clarke M., Fenton M., Forbes C., Glanville J., Hicks N.J., Moody J. (2006). How to formulate research recommendations. BMJ.

[15] Iannotti F., Prati P., Fidanza A., Iorio R., Ferretti A., Pèrez Prieto D., Kort N., Violante B., Pipino G., Schiavone Panni A. (2020). Prevention of Periprosthetic Joint Infection (PJI): A Clinical Practice Protocol in High-Risk Patients. Trop. Med. Infect. Dis..

[16] Liu H., Xing H., Zhang G., Wei A., Chang Z. (2025). Risk factors for surgical site infections after orthopaedic surgery: A meta-analysis and systematic review. Int. Wound J..

[17] Liu H., Wang Y., Xing H., Chang Z., Pan J. (2024). Risk factors for deep surgical site infections following orthopedic trauma surgery: A meta-analysis and systematic review. J. Orthop. Surg. Res..

[18] Kong L., Liu Z., Meng F., Shen Y. (2017). Smoking and Risk of Surgical Site Infection after Spinal Surgery: A Systematic Review and Meta-Analysis. Surg. Infect..

[19] Shao J., Chang H., Zhu Y., Chen W., Zheng Z., Zhang H., Zhang Y. (2017). Incidence and risk factors for surgical site infection after open reduction and internal fixation of tibial plateau fracture: A systematic review and meta-analysis. Int. J. Surg..

[20] Zhang Y., Zheng Q.J., Wang S., Zeng S.X., Zhang Y.P., Bai X.J., Hou T.Y. (2015). Diabetes mellitus is associated with increased risk of surgical site infections: A meta-analysis of prospective cohort studies. Am. J. Infect. Control.

[21] Goldman A.H., Tetsworth K. (2023). AAOS Clinical Practice Guideline Summary: Prevention of Surgical Site Infection After Major Extremity Trauma. J. Am. Acad. Orthop. Surg..

[22] Onggo J.R., Onggo J.D., de Steiger R., Hau R. (2020). Greater risks of complications, infections, and revisions in the obese versus non-obese total hip arthroplasty population of 2,190,824 patients: A meta-analysis and systematic review. Osteoarthr. Cartil..

[23] Indelli P.F., Iannotti F., Ferretti A., Valtanen R., Prati P., Pérez Prieto D., Kort N.P., Violante B., Tandogan N.R., Schiavone Panni A. (2022). Recommendations for periprosthetic joint infections (PJI) prevention: The European Knee Associates (EKA)-International Committee American Association of Hip and Knee Surgeons (AAHKS)-Arthroplasty Society in Asia (ASIA) survey of members. Knee Surg. Sports Traumatol. Arthrosc..

[24] Choe H., Indelli P.F., Ricciardi B., Kim T.Y., Homma Y., Kigera J., Veloso Duran M., Khan T. (2025). What Are the Absolute Contraindications for Elective Total Knee or Hip Arthroplasty?. J. Arthroplast..

[25] Seidelman J.L., Mantyh C.R., Anderson D.J. (2023). Surgical Site Infection Prevention: A Review. JAMA.

[26] Chen Y., Chen W. (2024). Association between malnutrition status and total joint arthroplasty periprosthetic joint infection and surgical site infection: A systematic review meta-analysis. J. Orthop. Surg. Res..

[27] Moore J., Isler M., Barry J., Mottard S. (2014). Major wound complication risk factors following soft tissue sarcoma resection. Eur. J. Surg. Oncol..

[28] Sugita S., Hozumi T., Yamakawa K., Goto T., Kondo T. (2016). Risk factors for surgical site infection after posterior fixation surgery and intraoperative radiotherapy for spinal metastases. Eur. Spine J..

[29] Morii T., Ogura K., Sato K., Kawai A. (2024). Incidence and risk of surgical site infection/periprosthetic joint infection in tumor endoprosthesis-data from the nationwide bone tumor registry in Japan. J. Orthop. Sci..

[30] Edmiston C.E., Chitnis A.S., Lerner J., Folly E., Holy C.E., Leaper D. (2019). Impact of patient comorbidities on surgical site infection within 90 days of primary and revision joint (hip and knee) replacement. Am. J. Infect. Control.

[31] Zhao D., Liang G.H., Pan J.K., Zeng L.F., Luo M.H., Huang H.T., Han Y.H., Lin F.Z., Xu N.J., Yang W.Y. (2023). Risk factors for postoperative surgical site infections after anterior cruciate ligament reconstruction: A systematic review and meta-analysis. Br. J. Sports Med..

[32] Ko M.S., Choi C.H., Yoon H.K., Yoo J.H., Oh H.C., Lee J.H., Park S.H. (2021). Risk factors of postoperative complications following total knee arthroplasty in Korea: A nationwide retrospective cohort study. Medicine.

[33] Liukkonen R., Honkanen M., Skyttä E., Eskelinen A., Karppelin M., Reito A. (2024). Clinical Outcomes After Revision Hip Arthroplasty due to Prosthetic Joint Infection-A Single-Center Study of 369 Hips at a High-Volume Center with a Minimum of One Year Follow-Up. J. Arthroplast..

[34] Joanroy R., Gubbels S., Møller J.K., Overgaard S., Varnum C. (2024). Risk of second revision and mortality following first-time revision due to prosthetic joint infection after primary total hip arthroplasty: Results on 1,669 patients from the Danish Hip Arthroplasty Register. Acta Orthop..

[35] Cheng H., Chen B.P., Soleas I.M., Ferko N.C., Cameron C.G., Hinoul P. (2017). Prolonged Operative Duration Increases Risk of Surgical Site Infections: A Systematic Review. Surg. Infect..

[36] Randelli P.S., Merghani K., Atilla B., Efimov D., El Hadj L.A., Garín Z.D.E., Lustig S., Mahajan R., Marazzi F.C., Menon A. (2025). 2025 ICM: Surgical Procedure Time. J. Arthroplast..

[37] Martins L.P., Ruggieri P., Parwani R., Emara K.M., Angelini A. (2025). Bone and Joint Infection in Immunocompromised Patients. Bone and Joint Infections.

[38] Abbaszadeh A., Yilmaz M.K., Izadi N., Hoveidaei A.H., Taheriazam A., Abedi A.A., Parvizi J. (2026). Efficacy of Debridement, Antibiotics, and Implant Retention in Total Hip and Knee Arthroplasty: A Systematic Review and Meta-Analysis. J. Arthroplast..

[39] Hansen E., Ji B., Dietz M.J., Hoveidaei A.H., Zahar A., Mu W., Shahi A., Bozhkova S.A., Abolghasemian M., Angad C. (2025). 2025 ICM: One-Stage Exchange. J. Arthroplast..

[40] Elhence A., Böhler C., Kolhoff F., Fraval A., Sharma R.K., Belden K., Aggarwal V.K., Amanatullah D., Ascione T., Atilla B. (2025). 2025 ICM: Two-Stage. J. Arthroplast..

[41] Klasan A., Dworschak P., Heyse T.J., Malcherczyk D., Peterlein C.D., Schüttler K.F., Lahner M., El-Zayat B.F. (2018). Transfusions increase complications and infections after hip and knee arthroplasty: An analysis of 2760 cases. Technol Health Care.

[42] CDC National and State Healthcare-Associated Infections Progress Report, Published April 2024. https://www.cdc.gov/healthcare-associated-infections/php/data/progress-report.html.

[43] Yang Q., Sun J., Yang Z., Rastogi S., Liu Y.F., Zhao B.B. (2024). Evaluation of the efficacy of chlorhexidine-alcohol vs. aqueous/alcoholic iodine solutions for the prevention of surgical site infections: A systematic review and meta-analysis. Int. J. Surg..

[44] Wade R.G., Burr N.E., McCauley G., Bourke G., Efthimiou O. (2021). The Comparative Efficacy of Chlorhexidine Gluconate and Povidone-iodine Antiseptics for the Prevention of Infection in Clean Surgery: A Systematic Review and Network Meta-analysis. Ann. Surg..

[45] Cai Y., Xu K., Hou W., Yang Z., Xu P. (2017). Preoperative chlorhexidine reduces the incidence of surgical site infections in total knee and hip arthroplasty: A systematic review and meta-analysis. Int. J. Surg..

[46] Ritter B., Herlyn P.K.E., Mittlmeier T., Herlyn A. (2020). Preoperative skin antisepsis using chlorhexidine may reduce surgical wound infections in lower limb trauma surgery when compared to povidone-iodine—A prospective randomized trial. Am. J. Infect. Control.

[47] Hanish S.J., Kirwan M.J., Hou N., Coble T.J., Mihalko W.M., Holland C.T. (2025). Surgical Site Preparation Using Alcohol with Chlorhexidine Compared with Povidone Iodine with Chlorhexidine Results in Similar Rate of Infection After Primary Total Joint Arthroplasty. Antibiotics.

[48] Groenen H., Bontekoning N., Jalalzadeh H., Buis D.R., Dreissen Y.E., Goosen J.H., Graveland H., Griekspoor M., IJpma F.F., Van Der Laan M.J. (2024). Incisional Wound Irrigation for the Prevention of Surgical Site Infection: A Systematic Review and Network Meta-Analysis. JAMA Surg..

[49] Thom H., Norman G., Welton N.J., Crosbie E.J., Blazeby J., Dumville J.C. (2021). Intra-Cavity Lavage and Wound Irrigation for Prevention of Surgical Site Infection: Systematic Review and Network Meta-Analysis. Surg. Infect..

[50] Egerci O.F., Yapar A., Dogruoz F., Selcuk H., Kose O. (2024). Preventive strategies to reduce the rate of periprosthetic infections in total joint arthroplasty; a comprehensive review. Arch. Orthop. Trauma. Surg..

[51] Guerra-Farfán E., Najafi F., Hamidreza Y., Randelli P.S., Albelooshi A., Alenezi H., Burgo F.J., Devito F.S., Ekhtiari S., Federica R. (2025). 2025 ICM: Surgical Site Irrigation. J Arthroplast..

[52] Seta J.F., Weaver M.J., Hallstrom B.R., Zheng H.T., Larese D.M., Dailey E.A., Markel D.C. (2025). Intraoperative Irrigation and Topical Antibiotic Use Fail to Reduce Early Periprosthetic Joint Infection Rates: A Michigan Arthroplasty Registry Collaborative Quality Initiative Study. J. Arthroplast..

[53] Dudek B., Brożyna M., Karoluk M., Frankiewicz M., Migdał P., Szustakiewicz K., Matys T., Wiater A., Junka A. (2024). In vitro and in vivo translational insights into the intraoperative use of antiseptics and lavage solutions against microorganisms causing orthopedic infections. Int. J. Mol. Sci..

[54] Woodmansey E., Buttacavoli F.A., Riesgo A., Bibbo C., Tedesco N., Rodriguez D., Lebby E., Danoff J.R., Carli A.V. (2025). Intraoperative Wound Irrigation in Orthopaedic Surgery: A Survey of Current Understanding and Practice Across the United States. Arthroplast. Today.

[55] Koburger T., Hübner N.O., Braun M., Siebert J., Kramer A. (2010). Standardized comparison of antiseptic efficacy of triclosan, PVP-iodine, octenidine dihydrochloride, polyhexanide and chlorhexidine digluconate. J. Antimicrob. Chemother..

[56] Rippon M.G., Rogers A.A., Ousey K. (2023). Polyhexamethylene biguanide and its antimicrobial role in wound healing: A narrative review. J. Wound Care.

[57] Bianchi T., Wolcott R.D., Peghetti A., Leaper D., Cutting K., Polignano R., Rosa Rita Z., Moscatelli A., Greco A., Romanelli M. (2016). Recommendations for the management of biofilm: A consensus document. J. Wound Care.

[58] Wood T., Ekhtiari S., Mundi R., Citak M., Sancheti P.K., Guerra-Farfan E., Schemitsch E., Bhandari M. (2020). The Effect of Irrigation Fluid on Periprosthetic Joint Infection in Total Hip and Knee Arthroplasty: A Systematic Review and Meta-Analysis. Cureus.

[59] Wolfhagen N., Boldingh Q.J.J., Boermeester M.A., de Jonge S.W. (2022). Perioperative care bundles for the prevention of surgical-site infections: Meta-analysis. Br. J. Surg..

[60] National Guideline System Website (PNLG). https://siot.it/wp-content/uploads/2021/06/LG-366-SIOT-Prevenzione-delle-infezioni-in-chirurgia-ortopedica.pdf.

[61] Zhu Z., Tung T.H., Su Y., Li Y., Luo H. (2025). Intrawound vancomycin powder for prevention of surgical site infections in primary joint arthroplasty: An umbrella review of systematic reviews and meta-analyses. Int. J. Surg..

[62] Dietz M.J., Choe H., Abedi A.A., Austin M.S., Bingham J., Bouji N., Clyburn T.A., Hieda Y., Lizcano J.D., Lora-Tamayo J. (2025). 2025 ICM: Antibiotics Usage Criteria. J. Arthroplast..

[63] Surgical Site Infection Event. NHSN, January 2024. https://www.cdc.gov/nhsn/pdfs/pscmanual/9pscssicurrent.pdf.

[64] Wildeman P., Rolfson O., Wretenberg P., Nåtman J., Gordon M., Söderquist B., Lindgren V. (2024). Effect of a national infection control programme in Sweden on prosthetic joint infection incidence following primary total hip arthroplasty: A cohort study. BMJ Open.

